# A meta-analysis of safety and efficacy of endovascular aneurysm repair in aneurysm patients with severe angulated infrarenal neck

**DOI:** 10.1371/journal.pone.0264327

**Published:** 2022-02-24

**Authors:** Giulia Bernardini, Sarah Litterscheid, Giovanni Battista Torsello, Giovanni Federico Torsello, Efthymios Beropoulis, Denise Özdemir-van Brunschot

**Affiliations:** 1 Department of Vascular Surgery and Organ Transplant Unit, University Hospital of Catania, Catania, Italy; 2 Institute for Vascular Research, St Franziskus Hospital, Münster, Germany; 3 Department of Diagnostic and Interventional Radiology, Charité University Medicine, Berlin, Germany; 4 Department of Vascular and Endovascular Therapy, Augusta Hospital and Catholic Hospital Group, Düsseldorf, Germany; NIHR Leicester Biomedical Research Centre, UNITED KINGDOM

## Abstract

**Objectives:**

A growing number of abdominal aortic aneurysms with severe angulated neck anatomy is treated by endovascular means. However, contradictory early and late outcomes have been reported. Our review and outcome analysis attempted to evaluate the available literature and provide clinicians with a base for clinical implementation and future research.

**Materials and methods:**

A systematic review of the literature was undertaken to identify the outcomes of endovascular aneurysm repair in patients with severe infrarenal neck angulation (SNA **≥** 60°) vs non-severe neck angulation (NSNA). Outcome measures included perioperative complications, type 1a endoleak, neck-related secondary procedures, stent graft migration, aneurysm rupture, increase (>5mm) in sac diameter, all-cause and aneurysm-related mortality (PROSPERO Nr.: CRD42021233253).

**Results:**

Six observational studies reporting on 5981 patients (1457 with SNA and 4524 with NSNA) with a weighted mean follow-up period of 1.8 years were included. EVAR in SNA compared with NSNA was associated with a higher rate of type 1a endoleak at 30 days (4.0% *vs* 1.8%; p< 0.00001), at 1 year (2.8% *vs* 1.9%; p<0.03), at 2 years (4.9% *vs* 2.1%; p< 0.0002), at 3 years (5.6% *vs* 2.6%; p< 0.0001). The rate of neck-related secondary procedures was significantly higher at 1 year (6.6% vs 3.9%; p<0.05) and at 3 years (13.1% vs 9%; p<0.05). Graft migration, aneurysm sack increase, aneurysm rupture and all-cause mortality were not statistically different at mid-term.

**Conclusions:**

The use of EVAR in severely angulated infrarenal aortic necks is associated with a high rate of early and mid-term complications. However, aortic related and all-causes mortality are not higher compared to patients with NSNA. Therefore, EVAR should be cautiously used in patients with SNA.

## Introduction

Endovascular aortic repair (EVAR) of abdominal aortic aneurysms (AAA) with severe angulated infrarenal necks is point of discussion since its introduction as a feasible procedure [1].

Infrarenal aortic angulation has a negative impact on proximal graft fixation and in patients with severe neck angulation (SNA) it can lead to type 1a endoleak [2–4]. Adjunctive procedures including an aortic extension, bare metal stent (BMS), or endoanchors are used intra-operatively to avoid or treat a type 1a endoleak while fenestrated grafts or chimney’s may be used to treat a type 1a endoleak postoperatively [5]. Other suprarenal solutions like use of fenestrated grafts and the chimney technique have been described for treating persistent type 1a endoleak.

Often, proximal aortic neck angulation is evaluated as one of several hostile neck criteria but rarely as stand-alone risk factor in severe angulated proximal neck. To our knowledge only a few studies with small sample sizes and with conflicting results have been published [6–11].

Considering the lack of systematic evaluations on this specific topic, the aim of this meta-analysis was to analyse the influence of severe infrarenal neck angulation as main hostile neck parameter on the short and mid-term outcome after EVAR.

## Materials and methods

### Search strategy and selection criteria

Objectives, methodology of systematic review, and inclusion criteria for study enrollment were specified and documented in a protocol, registered in the International Prospective Registry of Systematic Reviews (PROSPERO: CRD42021233253). The review was performed according to the PRISMA (Preferred Reporting Items for Systematic reviews and Meta-Analyses) guidelines [12].

A systematic literature search was conducted in PubMed, Cochrane Central and Scopus including articles from January 2000 until February 2021. The following Medical Subject Headings (MeSH) algorithm was used: (angulated neck OR hostile neck) AND aortic aneurysm. The search was conducted by two independent investigators (GB and SL) and any disagreement was resolved by a third investigator (DÖ). Data were recorded in a web-based specialized software [13].

Studies concerning EVAR comparing patients with severe neck angulation (SNA) with patients with a non-severe neck angulation (NSNA) were considered eligible. SNA was defined as an angle ≥ 60° of intersection between lines of the long axis of the aneurysm and the long axis of the infrarenal neck.

The predefined inclusion criteria were full text English written studies, publications from January 2000 to February 2021, single center or multicenter, randomized control studies and retrospective comparative studies. Case series with less than 5 patients pro study arm were excluded. Exclusion criteria included dissected, ruptured, or mycotic AAA, primary treatment with open surgery or fenestrated and branched endovascular treatment.

For each included study we extracted year of publication, single or multi center design, first author, study design, total number of patients and number of patients in each treatment arm. Demographic characteristics and accessory hostile parameters were extracted. Both suprarenal and infrarenal fixation devices were included. Need of adjunctive procedures at proximal aortic neck, defined as chimney EVAR (ch-EVAR), use of BMS, endovascular suture by EndoAnchors (ESAR) were also extracted.

The quality of non-randomized trials was assessed according to the Newcastle-Ottawa Scale (NOS). This scale was developed to assess the quality of studies using a “star system” (maximum nine stars), in which a study is judged on three broad perspectives: (1) the selection of the study groups, (2) the comparability of the groups, and (3) the ascertainment of outcome of interest [14].

### Endpoints

Outcome measures included perioperative complications, early and late type 1a endoleak, neck-related secondary procedures, stent graft migration, increase (>5mm) in sac diameter, aneurysm rupture, aneurysm-related and all-cause mortality, according to the reporting standards [15].

### Statistical analysis

The meta-analysis was performed using Review Manager (version 5.4 The Cochrane Collaboration, Oxford, UK). Data were pooled using the random effects model, as proposed by DerSimonian and Laird, and presented using odds ratio (OR) and 95% confidence interval (CI) [16]. To assess for heterogeneity, the I^2^ statistic was used. A I^2^ > 75% was used as a threshold in indicating significant heterogeneity. In cause of heterogeneity, reasons were explored. Funnel plots were used to assess publication bias. A p value ≤ 0.05 was considered significant.

## Results

### Study characteristics and quality assessment

**Fig 1:**
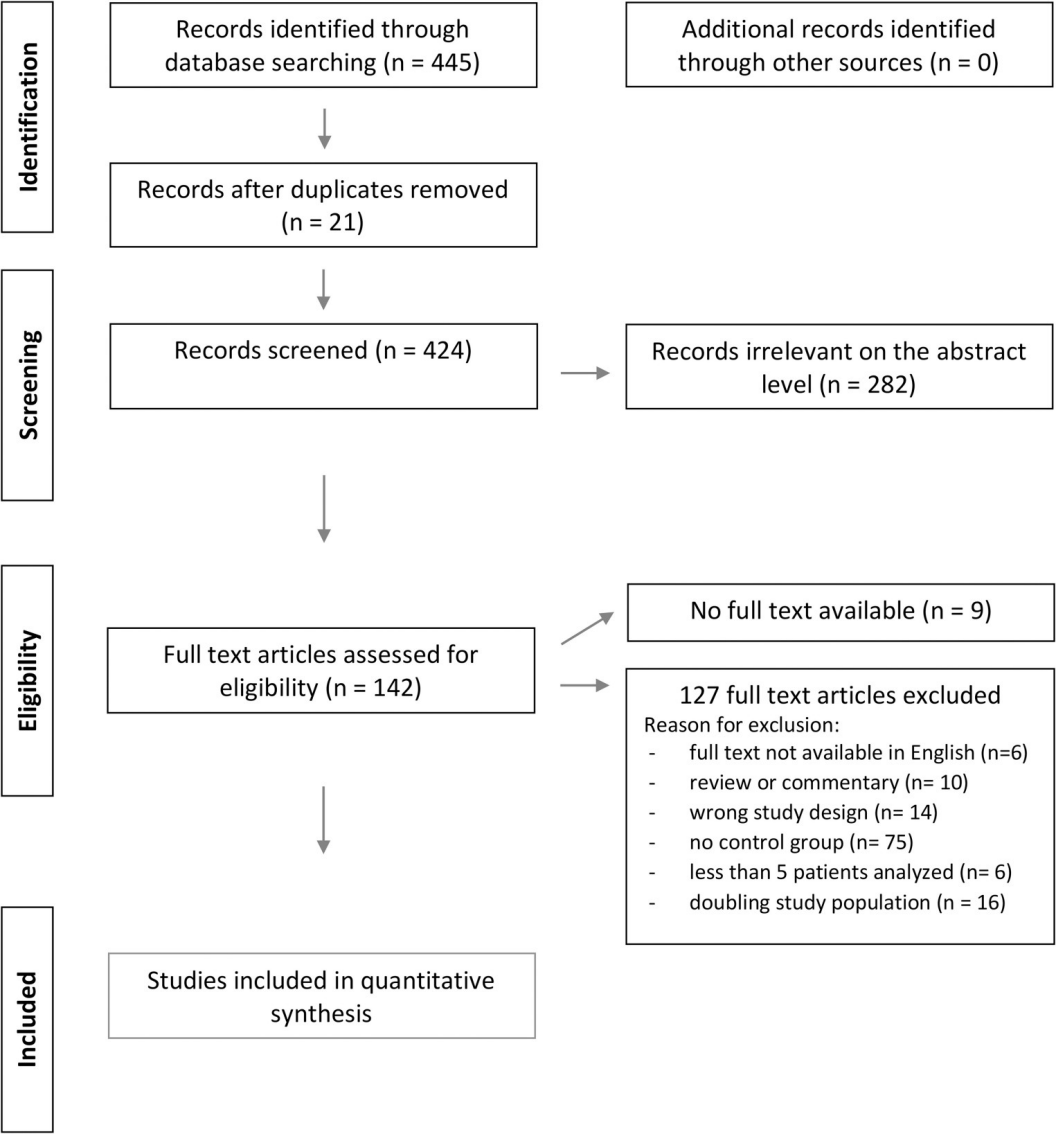
Prisma flow chart.

Six studies of initially 445 publications retrieved from our data base search fulfilled the inclusion criteria [6–11]. (Fig 1) The selected publications reported on the outcome of 5981 patients who underwent EVAR for AAA of which 1457 SNA patients presented with an infrarenal angle **≥** 60° and 4524 patients with NSNA. The follow-up period reached from 1 to 7 years with a weighted mean of 1.8 ± 2.4 years per followed patient.

Evaluation of analysed studies according to the modified Newcastle-Ottawa Scale revealed a high score of ≥ 6 for all included studies as presented in Table 1.

**Table 1:** Study characteristics.

Study	Total	SNA	NSNA	Adj. procedure/% of population	Type of endograft*	Mean FU** (years)	NOS
Chinsakchai et al., 2020 ^6^	198	54	144	Cuff or Palmaz/18.6%	Endurant II, Zenith, Gore Excluder	4.5	6
Hobo et al., 2007 ^7^	5183	1152	4031	-	Zenith, Talent, Gore Excluder	1.5	7
Le et al., 2016 ^8^	72	34	38	-	Zenith, Endurant, Gore Excluder, Seal	1.5	6
Malas et al., 2017 ^9^	218	151	67	Cuff /4%	Aorfix	5	7
Murray et al., 2020 ^10^	200	21	179	Cuff /14.3%	Treovance	1	7
Oliveira et al., 2018 ^11^	110	45	65	-	Endurant II	7	6
Total	5.981	1.457	4.524		Z: 43.7%, Ta: 30%, Ex: 15.3%, En: 3.8%, A: 3.6%, Tr: 3.4%, S: 0.2%		
**Weighted Mean**				0.3%	Suprarenal: 81.4% Infrarenal: 18.6%	1.8	6.5

* Aorfix® (Lombard Medical, Didcot, UK).

Endurant II® (Medtronic Cardiovascular, Santa Rosa, CA, USA).

Gore Excluder® (WL Gore & Associates, W.L. Gore Inc, Flagstaff, AZ, USA).

Seal ® (S&G Biotech, Seongnam, Korea).

Talent® (Medtronic Cardiovascular, Santa Rosa, CA, USA).

Treovance® (Terumo Aortic, Sunrise, FL, USA).

Zenith® (Cook Medical, Bloomington, IN, USA).

** Calculation in relation to the study population of included studies.

Adj = adjunctive; FU = follow up; NOS = Newcastle-Ottawa Scale; Z = Zenith®; Ta = Talent®; Ex = Excluder®; En = Endurant®; A = Aorfix®, Tr = Treovance®, S = Seal®.

### Demographics

Demographics and comorbidities of the study populations are depicted in Table 2. Mean age was 2.2 years higher in the SNA population (74.5±7.6 *vs* 72.3±8.1 years in NSNA). ASA III-IV classification and COPD were more frequent in the SNA patient population. Additional data for each study are shown in Table 2.

**Table 2:** Demographics and comorbidities.

	Chinsakchai et al., 2020		Hobo et al., 2007		Le et al., 2016		Malas et al., 2017		Murray et al., 2020		Oliveira et al., 2018		Mean	
	**Severe Neck Angulation** versus **Non-Severe Neck Angulation**													
**Number of patients**	54	144	1152	4031	34	38	151	67	179	21	45	65		
**Mean age (years)**	77.5	74.7	74.3	72.1	75.6	72.3	76.3	74.0	73.0	72.6	75.6	72.7	74.5	72.3
**Female sex (%)**	29.6	18.7	9.7	5.2	29	5	35	15	4.8	7.2	20	9.2	11.5	6.1
**Hypertension (%)**	77.8	77.8	65.5	66.4	70	76	83	90	81	78.2	55.6	53.8	66.9	67.9
**Diabetes mellitus (%)**	16.7	20.1	12.3	13.1	32	32	17	19	19	20.1	13.3	23.1	13,1	14.2
**Coronary artery disease (%)**	35.2	28.5	61.6	60.8	26	29	44	51	19	38	48.9	41.5	58.0	57.9
**Dyslipidemia (%)**	29.6	25	45.6	45.9	41	42	-	-	28.6	38	-	-	44.4	44.8
**Cerebrovascular disease (%)**	5.6	8.3	-	-	21	21	-	-	-	-	8.9	18.5	9.5	13.7
**Smoking (%)**	-	-	23.2	22.6	35	45	83	97	71.4	62	78.5	78.5	28.3	28.1
**Chronic pulmonary obstructive disease (%)**	11.1	14.6	45	41.5	-	-	33	28	9.5	18	31.1	20	42.0	38.9
**Cardiovascular risk factor (%)**	3.7	14.6	19.6	19.5	12	8	15	13	28.6	15.1	35.6	30.8	19.4	19.0
**American Society of Anesthesiologists III-IV (%)**	81.5	79.2	55	47.2	-	-	-	-	66.6	57.6	73.3	66.2	56.7	49.1

For each study considered, the first column (in blue) depicts Severe Neck Angulation group and the second column Non-Severe Neck Angulation group. Missing values are marked with (-).

### Perioperative complications

Four studies reported on perioperative complications comparing the rates for both SNA and NSNA groups [6–11]. With 16.2% there was a higher rate in the SNA than in the NSNA with 7.3% but the difference was not significant (p<0.08).

### Type 1a endoleak

**Fig 2:**
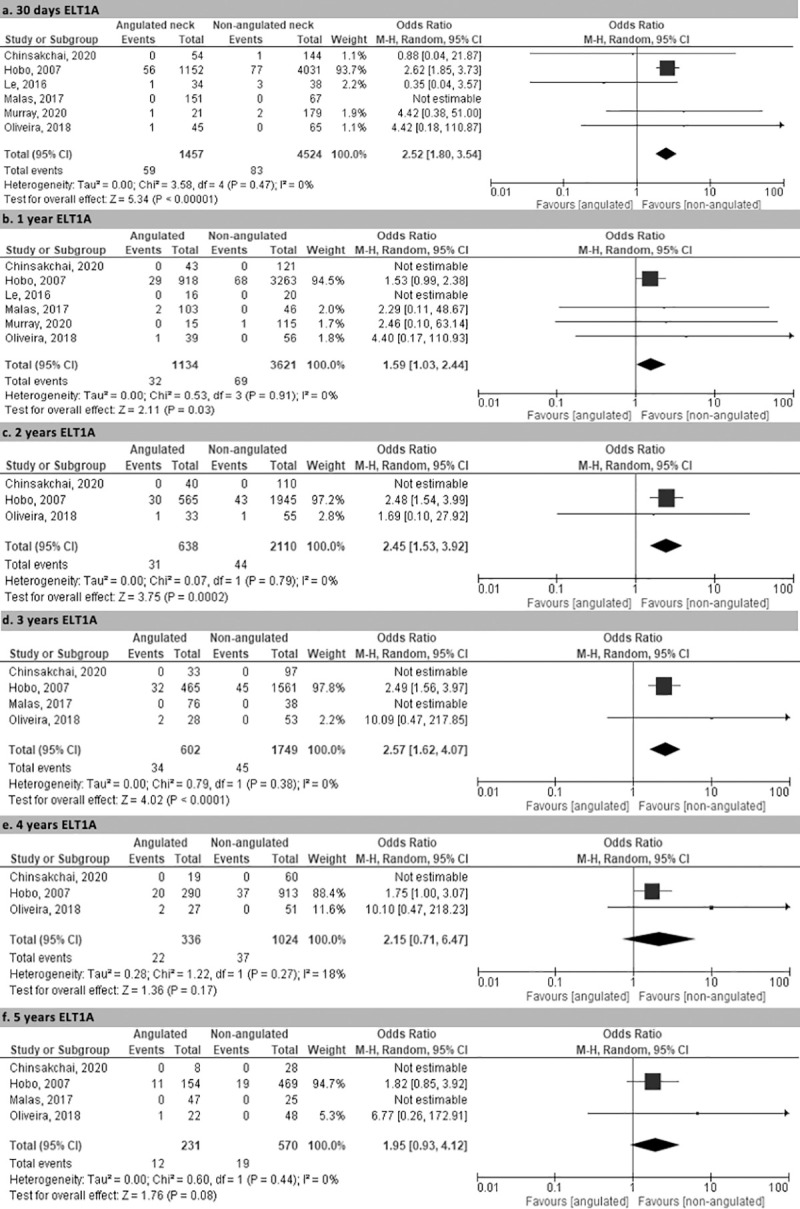
Rate of endoleak type 1A. Rate of endoleak type 1A at 30 days (a), 1 year (b), 2 years (c), 3 years (d), 4 years (e) and 5 years (f).

The rate of early type 1a endoleak was reported in all selected studies and was significantly higher in the SNA group at 30 days (4.0% vs. 1.8%; p< 0.00001; OR 2.52 95% CI 1.80–3.54) (Fig 2A). The rate of type 1a endoleak was significantly higher in the SNA group at 1 year (2.8% vs 1.9%; p< 0.03; OR 1.59 95% CI 1.03–2.44), at 2 years (4.9% *vs* 2.1%; p< 0.0002; OR 2.45 95% CI 1.53–3.92) and at 3 years (5.6% *vs* 2.6%; p< 0.00001; OR 2.57 95% CI 1.62–4.07). At 4 and 5 years type 1a endoleak was higher but not statistically significant (at 4 years 6.5% *vs* 3.6%; p< 0.17; at 5 years 5.2% *vs* 3.3%; p< 0.08; Fig 2B–2F).

### Neck-related secondary procedures

**Fig 3:**
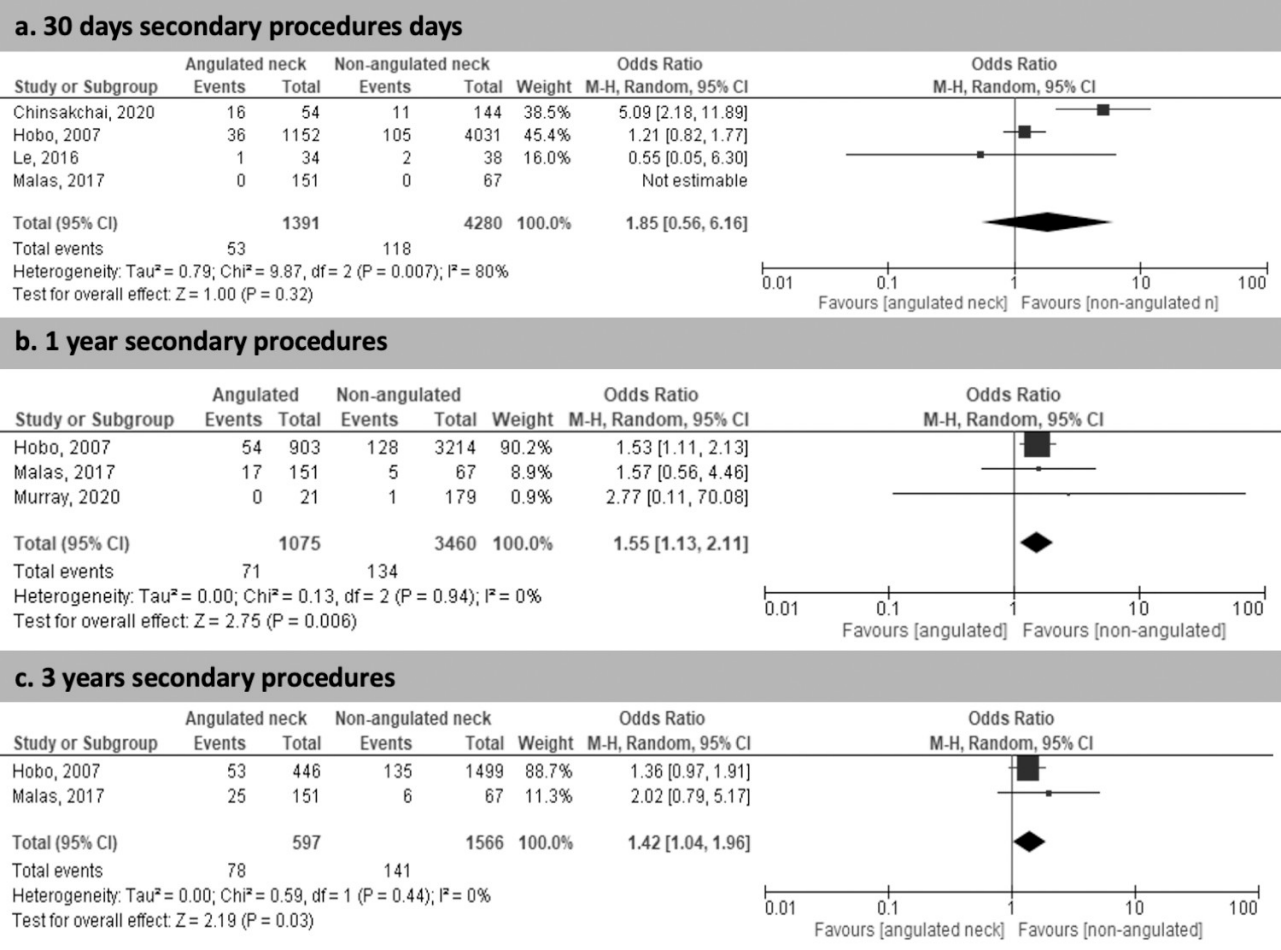
Rate of neck related secondary procedures. Rate of neck related secondary procedures at 30 days (a), 1 year (b) and 2 years (c).

The rate of neck-related secondary procedures was higher in the SNA group at 1 year (6.6% vs 3.9%; p< 0.05; OR 1.55 95% CI 1.13–2.11) and at 3 years (13.1% vs 9%; p<0.05; OR 1.42 95% CI 1.04–1.96). (Fig 3A–3C) Data regarding longer follow-up were only presented in the study by Malas *et al* [9].

### Migration

Migration rates at 30 days (1.4% *vs* 0.8%; p< 0.05; OR 1.88 95% CI 1.07–3.30) (S1 Fig) and at 1 year (5.4% *vs* 4.0%; p< 0.05; OR 1.41 95% CI 1.03–1.94) (S2 Fig) were significantly higher in the SNA group. At 2,3 and 5 years migration rates were not statistically significant.

### Aneurysm sac increase and rupture

At 1 year no difference in sac increase between the groups (1.8 *vs* 1.7%) was detected. Reported aneurysm rupture was rare and without changes between the groups from 30 days to 5 years. (S3 Fig).

### Aneurysm-related and all-cause mortality

Aneurysm-related mortality was significantly higher in the SNA group at 1 year (6.4% vs. 4.3%; p< 0.05; OR 1.51 95% CI 1.16–1.98) but statistically not different at the other time points. (S4 Fig) No statistically different all-cause mortality rate was depicted from 30 days to 5 years. (S5 Fig).

A summary of outcomes is described in Table 3.

**Table 3:** Summary of outcomes.

Outcome measure	Number of studies	Number of cases	OR	95% CI
*Peri-operative complications*	4	54	2.45	0.91–6.59
*EL1A*				
at 30 days	5	5981	2.52	1.80–3.54
at 6 months	3	497	0.90	0.16–5.02
at 1 year	4	4755	1.59	1.03–2.44
at 2 years	2	2748	2.45	1.53–3.92
at 3 years	2	2351	2.57	1.62–4.07
at 4 years	2	1360	2.15	0.71–6.47
at 5 years	2	801	1.95	0.93–4.12
*Neck-related secondary procedures*				
at 30 days	3	5671	1.85	0.56–6.16
at 1 year	3	4535	1.55	1.13–2.11
at 3 years	2	2163	1.42	1.04–1.96
Migration				
at 30 days	2	5783	1.88	1.07–3.30
at 1 year	2	4611	1.41	1.03–1.94
at 2 years	2	2650	1.62	0.83–3.19
at 3 years	2	2177	1.60	0.92–2.76
at 5 years	3	804	1.16	0.52–2.57
*Sac increase at 1 year*	2	395	2.05	0.34–12.45
*Aneurysm rupture*				
at 30 days	2	5981	2.40	0.11–53.45
at 6 months	3	723	0.82	0.08–8.53
at 1 year	2	5765	0.85	0.08–9.31
at 2 years	4	2959	1.00	0.34–2.93
at 3 years	4	2414	1.51	0.88–2.60
at 4 years	2	1484	1.25	0.66–2.38
*Aneurysm-related mortality*				
at 6 months	2	325	0.94	0.15–6.07
at 1 year	3	5673	1.51	1.16–1.98
at 2 years	2	267	1.33	0.32–5.59
at 3 years	2	237	1.81	0.45–7.31
at 4 years	2	206	2.50	0.45–13.80
at 5 years	2	138	1.61	0.27–9.67
*All-cause mortality*				
at 30 days	4	5781	1.20	0.64–2.25
at 1 year	5	5861	1.04	0.90–1.21
at 2 years	3	460	1.55	0.87–2.77
at 3 years	3	434	1.64	0.99–2.73
at 4 years	3	380	1.74	1.04–2.91
at 5 years	3	332	1.56	0.93–2.61

All meta-analyses were performed with random effects mode.

## Discussion

This meta-analysis shows that EVAR for AAA with severely angulated neck is associated with higher rate of type 1a endoleak and need for neck-related reinterventions.

The growing experience in EVAR and the introduction of improved technologies encouraged the expansion of indications, especially for patients at significant risk for open surgery [17,18]. However, the liberal adoption of EVAR in hostile neck anatomies increases the risk of endoleak. AbuRahma et al. reported high rate of endoleak in their patients with SNA [19]. Also Tsilimparis et al. suggested that infrarenal angulation is an independent predictor of secondary interventions [20]. Antoniou et al. found that hostile neck anatomy (HNA) was associated with a twofold increased risk of 30-day morbidity, a nine-fold increased risk of aneurysm-related mortality within 1 year, higher rate of proximal neck dilation, type 1a endoleak and reintervention [21]. Also our meta-analysis confirms the higher rate of type 1a endoleaks and secondary interventions within 1 year. Despite the high-risk profile of the SNA group patients, the aneurysm-related mortality and rupture did not show a statistical difference between severe angulated and non-angulated necks at mean follow up period. However, this might be explained by the low number of cases and limited follow up.

In the present meta-analysis patients in the SNA group had a higher incidence of COPD and a higher operative risk based on ASA classification. These results are in accordance with previous reports underling an association between the clinical status, ASA status and anatomic complexity of aorta [22,23].

The patient based mean follow-up period was 21.6 ± 29 months and only 3 studies had a follow up longer than 2 years [6,9,11]. Long-term results beyond five years were presented only by Oliveira et al [11]. After a median follow-up of 7.4 years freedom from type 1a endoleak was 86.1% in the SNA group vs 96.6.2% in the NSNA group. Their experience underlines the role of closed follow-up also on mid- and long-term treating patients with severe angulated neck anatomy.

Concerning quality assessment, no randomized controlled studies have been found; however, the methodological quality of four out of six multicenter studies included in this review was high as evaluated with the Newcastle Ottawa Scale [7,9–11]. Nevertheless, the use of different devices created some heterogeneity among the study populations examined. (Table 1) One of those (Talent®, Medtronic Cardiovascular, Santa Rosa, CA, USA) is not commercially available anymore and two (Zenith®, Cook Medical, Bloomington, IN, USA and Excluder® WL Gore & Associates, Flagstaff, AZ, USA) have been modified from earlier generation devices. Recent design modifications have been introduced to overcome limitations regarding proximal neck anatomy and thereby expanding indications [24]. The current suprarenal fixation platforms, Treovance® (Terumo Aortic, Sunrise, FLA, USA) and Endurant II® (Medtronic Cardiovascular, Santa Rosa, CA, USA) are currently indicated to treat infrarenal necks up to 75° [25,26]. Moreover, the indication is expanded up to 90° with infrarenal platforms as Anaconda® (Terumo Aortic, Glasgow, UK), Aorfix® (Lombard Medical, Didcot, UK) and Conformable C3 device® (WL Gore & Associates, Flagstaff, AZ, USA). However, the infrarenal neck length should be at least 15 mm [27–29].

Finally, alternative endovascular options like parallel endograft techniques or use of fenestrated endografts may be technically challenging to perform, and long-term outcomes in severely angulated necks are lacking [30].

Adjunctive fixation with EndoAnchor during primary repair has been reported in patients with hostile neck to improve endograft apposition to the outer aortic curve, thus increasing proximal seal length [5,31,32]. Chaudhuri et al. reported on an incidence of type 1a endoleak of 2.4% (1/42) without neck related interventions [33]. However, reports directly comparing SNA with and without EndoAnchor are still lacking, and long-term durability is not known.

## Limitations

The results of the present study should be interpreted in the context of some limitations. First, the paucity number of studies available should be considered. The population weight was not equally distributed, with one study counting with more than 80% of the study population [12]. Additionally, the small studies have wide confidence intervals. Second, in current literature details are missing regarding the distance between the lowest renal artery and the maximum infrarenal angulation. Severe infrarenal angulation just below the ostium of the renal arteries will be of greater influence on outcomes compared to the same angulation 40 mm below the take-off of the renal arteries. Third, a wide range of endoprostheses, with both supra (81.4%) and infrarenal (18.6%) fixation and different IFU was analyzed, affecting study heterogeneity.

## Conclusions

The use of infrarenal EVAR devices in severely angulated aortic necks is associated with a high rate of early and mid-term complications. However, aortic-related and all-cause mortality is not higher compared to patients with NSNA at mid-term. From the present analysis, it may be concluded that an accurate patient selection and a careful morphometric assessment in SNA patients should be recommended. Prospective, multicenter registries with long-term data are urgently needed to identify the best treatment option in patients presenting with an infrarenal AAA with severe neck angulation, considered to be fit for treatment.

## Supporting information

S1 PRISMA checklist (DOC)

S1 Fig Rate of migration at 30 days. (DOCX)

S2 Fig Rate of migration at 1 year. (DOCX)

S3 Fig Rate of aneurysm rupture at 3 years. (DOCX)

S4 Fig Rate of aneurysm related mortality at 5 years. (DOCX)

S5 Fig Rate of all causes mortality at 5 years. (DOCX)

S1 Dataset Data set and statistical analysis. (DOCX)

## Abbreviations

AAA: aortic abdominal aneurysm
CAD: coronary artery disease
CI: confidence interval
COPD: chronic obstructive pulmonary disease
CRF: chronic renal failure
CVD: cerebrovascular disease
DM: diabetes mellitus
ESAR: endosuture aneurysm repair
EVAR: endovascular aneurysm repair
HTN: hypertension
IFU: instructions for use
NSNA: non severe neck angulation
SNA: severely neck angulation
