# Supplementary material for: A meta-analysis of safety and efficacy of endovascular aneurysm repair in aneurysm patients with severe angulated infrarenal neck
Source: PLoS One. 2022 Feb 24;17(2):e0264327. doi: 10.1371/journal.pone.0264327 (PMC8870420; doi:10.1371/journal.pone.0264327)
Supplement: S1 PRISMA checklist — (DOC) [file pone.0264327.s001.doc]

| **Section/topic** | **#** | **Checklist item** | **Reported on page #** |
| --- | --- | --- | --- |
| **TITLE** | | |  |
| Title | 1 | A Meta-Analysis of safety and efficacy of EVAR in aneurysm patients with angulated and hyperangulated neck |  |
| **ABSTRACT** | | |  |
| Structured summary | 2 | Objectives: A growing number of abdominal aortic aneurysms with severe angulated neck anatomy is treated by endovascular means. However, contradictory early and late outcomes have been reported. Our review and outcome analysis attempted to evaluate the available literature and provide clinicians with a base for clinical implementation and future research.  Materials and Methods: A systematic review of the literature was undertaken to identify the outcomes of endovascular aneurysm repair in patients with severe infrarenal neck angulation (SNA ≥ 60°) vs non-severe neck angulation (NSNA). Outcome measures included perioperative complications, type 1a endoleak, neck-related secondary procedures, stent graft migration, aneurysm rupture, increase (>5mm) in sac diameter, all-cause and aneurysm-related mortality (PROSPERO Nr.: CRD42021233253).  Results: Six observational studies reporting on 5981 patients (1457 with SNA and 4524 with NSNA) with a weighted mean follow-up period of 1.8 years were included. EVAR in SNA compared with NSNA was associated with a higher rate of type 1a endoleak at 30 days (4.0% vs 1.8%; p< 0.00001), at 1 year (2.8% vs 1.9%; p<0.03), at 2 years (4.9% vs 2.1%; p< 0.0002), at 3 years (5.6% vs 2.6%; p< 0.0001), but not at 4 years (6.5% vs 3.6%; p<0.17, n.s.), and at 5 years (5.2% vs 3.3%; p<0.08, n.s.). The rate of neck-related secondary procedures was significantly higher at 1 year (6.6% vs 3.9%; p<0.05) and at 3 years (13.1% vs 9%; p<0.05) but not at 30 days (3.8% vs 2.8%; p<0.32). Graft migration, aneurysm sack increase, aneurysm rupture and all-cause mortality were not statistically different.  Conclusions: The use of EVAR in severely angulated infrarenal aortic necks is associated with a high rate of early and late complications. However, aortic related and all-causes mortality are not higher compared to patients with NSNA. Therefore, EVAR should be cautiously used in patients with SNA. |  |
| **INTRODUCTION** | | |  |
| Rationale | 3 | Infrarenal aortic angulation has a negative impact on proximal graft fixation and in patients with severe neck angulation (SNA) it can lead to type 1a endoleak. Often, proximal aortic neck angulation is evaluated as one of several hostile neck criteria but rarely as stand-alone risk factor in severe angulated proximal neck.  Considering the lack of systematic evaluations on this specific topic, the aim of this meta-analysis was to analyse the influence of severe infrarenal neck angulation as main hostile neck parameter on the short and long-term outcome after EVAR. |  |
| Objectives | 4 | P – patients with infrarenal aneurysms in hyperangulated neck anatomies (all ages, all sex, all comorbidities, but excluding dissected, ruptured and infected aneurysms and those due to genetic disorders)  I - EVAR of severe infrarenal neck angulation (SNA ≥ 60°) abdominal aneurysms with all stent-graft types (excluding fenestrations, branches and conversion to open repair), all secondary interventions and adjunctive procedures  C – comparison group: EVAR of infrarenal neck angulation < 60° abdominal aneurysms  O - short and long-term outcomes regarding perioperative complications, type 1a endoleak, neck-related secondary procedures, stent graft migration, aneurysm rupture, increase (>5mm) in sac diameter, all-cause and aneurysm-related mortality  S – systematic review |  |
| **METHODS** | | |  |
| Protocol and registration | 5 | Objectives, methodology of systematic review, and inclusion criteria for study enrollment were specified and documented in a protocol, registered in the International Prospective Registry of Systematic Reviews (PROSPERO) with the number CRD42021233253). The review was performed according to the PRISMA (Preferred Reporting Items for Systematic reviews and Meta-Analyses) guidelines. |  |
| Eligibility criteria | 6 | Studies concerning EVAR comparing patients with severe neck angulation (infrarenal angle ≥ 60° of intersection between lines of the long axis of the aneurysm and the long axis of the infrarenal neck) with patients presenting a non-severe neck angulation (NSNA) were considered eligible. |  |
| Information sources | 7 | PubMed, Cochrane Central and Scopus |  |
| Search | 8 | Systematic literature including articles from January 2000 until February 2021. The following Medical Subject Headings (MeSH) algorithm was used: (angulated neck OR hostile neck) AND aortic aneurysm |  |
| Study selection | 9 | The predefined inclusion criteria were full text English written studies, publications from January 2000 to February 2021, single center or multicenter, randomized control studies and retrospective comparative studies. Case series with less than 5 patients pro study arm were excluded. Exclusion criteria included dissected, ruptured, or mycotic AAA, primary treatment with open surgery or fenestrated and branched endovascular treatment. |  |
| Data collection process | 10 | For each included study we extracted year of publication, single or multi center design, first author, study design, total number of patients and number of patients in each treatment arm. Demographic characteristics and accessory hostile parameters were extracted. Both suprarenal and infrarenal fixation devices were included. Need of adjunctive procedures at proximal aortic neck, defined as chimney EVAR, use of bare metal stent, endovascular suture by EndoAnchors were also extracted. |  |
| Data items | 11 | Duplicated were removed |  |
| Risk of bias in individual studies | 12 | The search was conducted by two independent investigators (GB and SL) and any disagreement was resolved by a third investigator (DÖ). Data were recorded in a web-based specialized software. |  |
| Summary measures | 13 | Odds ratio (OR) and 95% confidence interval (CI) |  |
| Synthesis of results | 14 | The meta-analysis was performed using Review Manager (version 5.4 The Cochrane Collaboration, Oxford, UK). Data were pooled using the random effects model and presented using odds ratio (OR) and 95% confidence interval (CI). To assess for heterogeneity, the I2 statistic was used. A I2 > 75% was used as a threshold in indicating significant heterogeneity. In cause of heterogeneity, reasons were explored. Funnel plots were used to assess publication bias. A p value ≤ 0.05 was considered significant |  |

Page 1 of 2

| **Section/topic** | **#** | **Checklist item** | **Reported on page #** |
| --- | --- | --- | --- |
| Risk of bias across studies | 15 | Funnel plots were used to assess publication bias. A p value ≤ 0.05 was considered significant |  |
| Additional analyses | 16 | The quality of non-randomized trials was assessed according to the Newcastle-Ottawa Scale (NOS). This scale was developed to assess the quality of studies using a “star system” (maximum nine stars), in which a study is judged on three broad perspectives: (1) the selection of the study groups, (2) the comparability of the groups, and (3) the ascertainment of outcome of interest. |  |
| **RESULTS** | | |  |
| Study selection | 17 | Six observational studies of initially 445 publications retrieved from our data base search fulfilled the inclusion criteria (**Figure 1**). They reported on a total of 5981 patients (1457 with SNA and 4524 with NSNA) with a weighted mean follow-up period of 1.8 years |  |
| Study characteristics | 18 | **Table I** summarizes studies characteristics |  |
| Risk of bias within studies | 19 | In **Table I** NOS quantification is presented |  |
| Results of individual studies | 20 | **Table I** summarizes individual studies results |  |
| Synthesis of results | 21 | **Table IIII** summarizes the synthesis of the results |  |
| Risk of bias across studies | 22 | In **Figure 2, 3** and **Supplementary Material**´s Figures Funnel Plots are presented |  |
| Additional analysis | 23 | Subgroup analyses were not done |  |
| **DISCUSSION** | | |  |
| Summary of evidence | 24 | This meta-analysis shows that EVAR for AAA with severe angulated neck is associated with higher rate of type 1a endoleak and need for neck-related reinterventions. |  |
| Limitations | 25 | The results of the present study should be interpreted in the context of some limitations. First, the paucity number of studies available should be considered. Second, in current literature details are missing regarding the distance between the lowest renal artery and the maximum infrarenal angulation. Severe infrarenal angulation just below the ostium of the renal arteries will be of greater influence on outcomes compared to the same angulation 40 mm below the take-off of the renal arteries. Third, a wide range of endoprosthesis, with both supra (81.4%) and infrarenal (18.6%) fixation and different IFU was analyzed, affecting study heterogeneity. |  |
| Conclusions | 26 | A prudent patient selection and a careful morphometric assessment in SNA patients should be recommended. Prospective, multicenter, long-term studies will play a fundamental role in assessing the best solution for patients with a severe neck angulation. |  |
| **FUNDING** | | |  |
| Funding | 27 | This research did not receive any specific grant from funding agencies in the public, commercial, or not-for- profit sectors. |  |

*From:*  Moher D, Liberati A, Tetzlaff J, Altman DG, The PRISMA Group (2009). Preferred Reporting Items for Systematic Reviews and Meta-Analyses: The PRISMA Statement. PLoS Med 6(7): e1000097. doi:10.1371/journal.pmed1000097

For more information, visit: **www.prisma-statement.org**.

Page 2 of 2
