# Supplementary figures and images for: A meta-analysis of safety and efficacy of endovascular aneurysm repair in aneurysm patients with severe angulated infrarenal neck
Source: PLoS One. 2022 Feb 24;17(2):e0264327. doi: 10.1371/journal.pone.0264327 (PMC8870420; doi:10.1371/journal.pone.0264327)

**Suppl. Fig 1. Rate of migration at 30 days.**

**
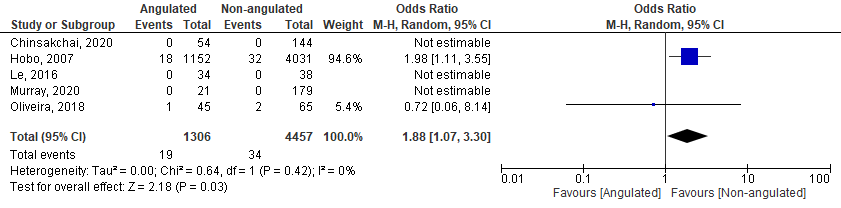
**

Supplement: S1 Fig — (DOCX) [file pone.0264327.s002.docx]

**Suppl. Fig 2. Rate of migration at 1 year.**

**
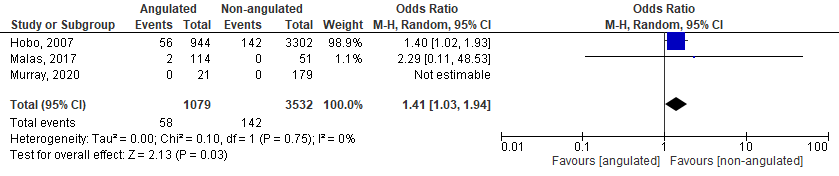
**

Supplement: S2 Fig — (DOCX) [file pone.0264327.s003.docx]

**Suppl. Fig 3. Rate of aneurysm rupture at 3 years.**

**
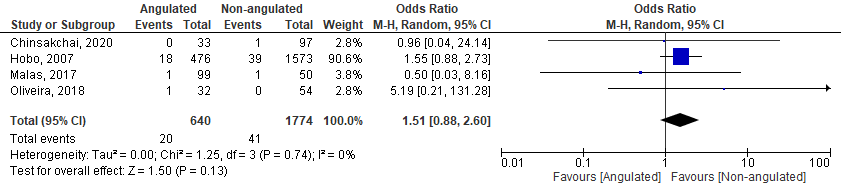
**

Supplement: S3 Fig — (DOCX) [file pone.0264327.s004.docx]

**Suppl. Fig 4. Rate of aneurysm related mortality at 5 years.**

**
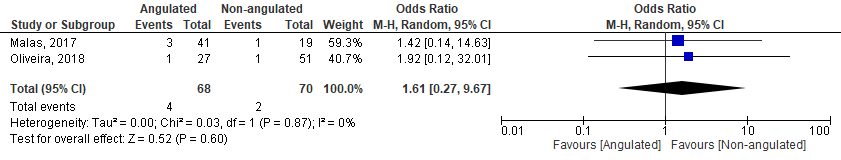
**

Supplement: S4 Fig — (DOCX) [file pone.0264327.s005.docx]

**Suppl. Fig 5. Rate of all causes mortality at 5 years.**

**
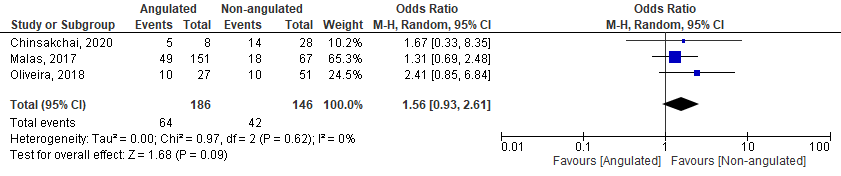
**

Supplement: S5 Fig — (DOCX) [file pone.0264327.s006.docx]
