# Supplementary material for: A meta-analysis of safety and efficacy of endovascular aneurysm repair in aneurysm patients with severe angulated infrarenal neck
Source: PLoS One. 2022 Feb 24;17(2):e0264327. doi: 10.1371/journal.pone.0264327 (PMC8870420; doi:10.1371/journal.pone.0264327)

**Peri-operative complications**


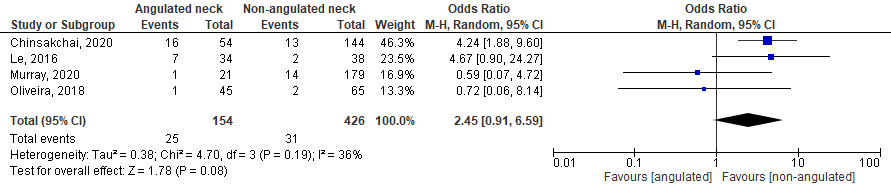


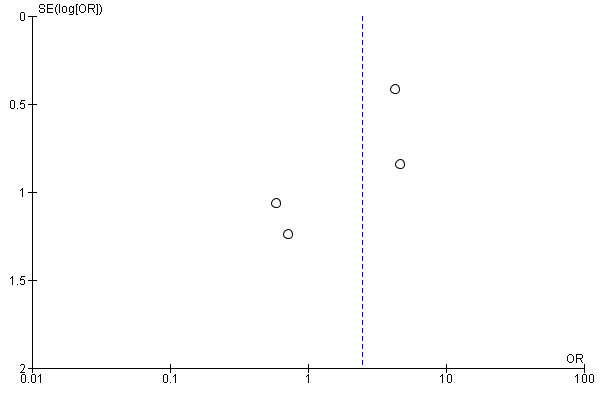


**Endoleaks at 30 days**

**
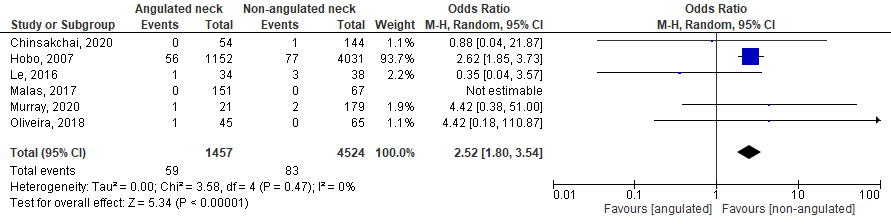
**

**
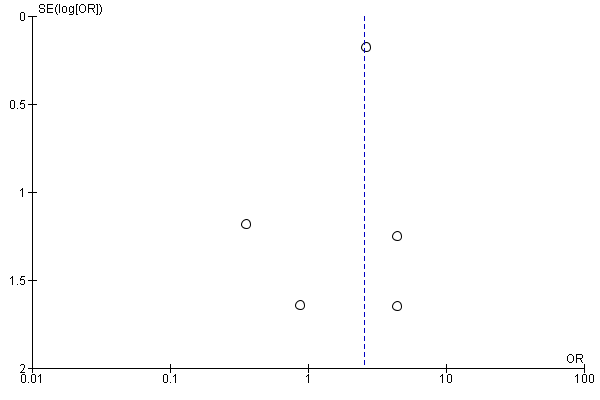
**

**Endoleaks at 6 months**

**
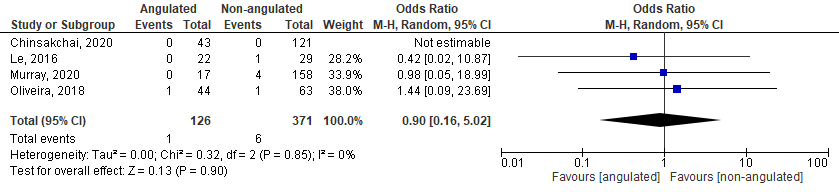
**

**
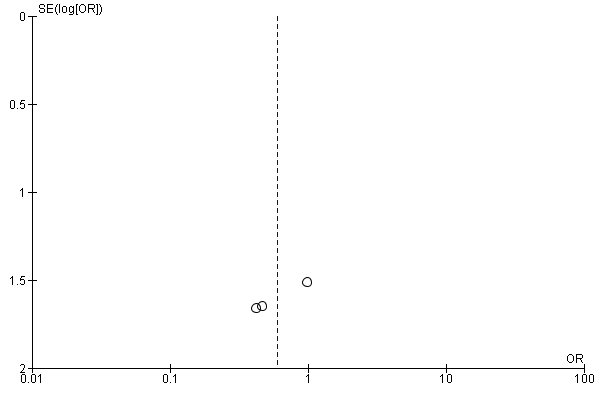
**

**Endoleaks at 1 year**

**
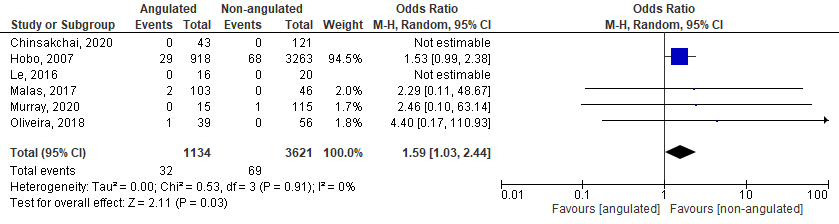
**

**
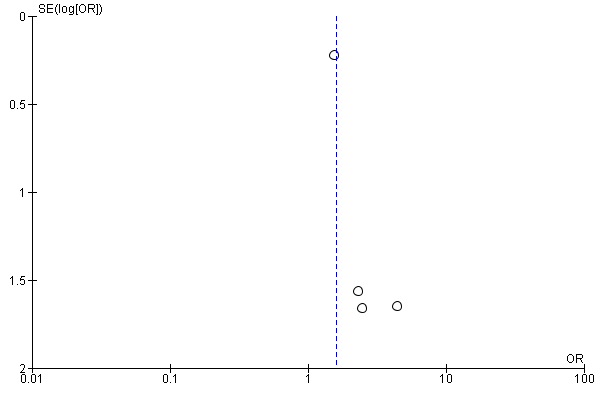
**

**Endoleaks at 2 years**


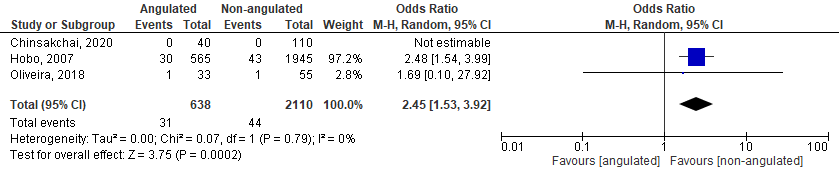


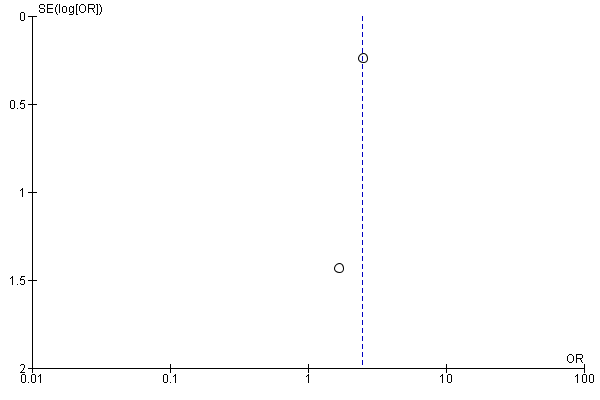


Endoleak at 3 years


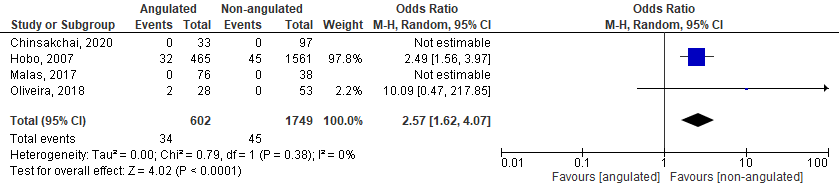


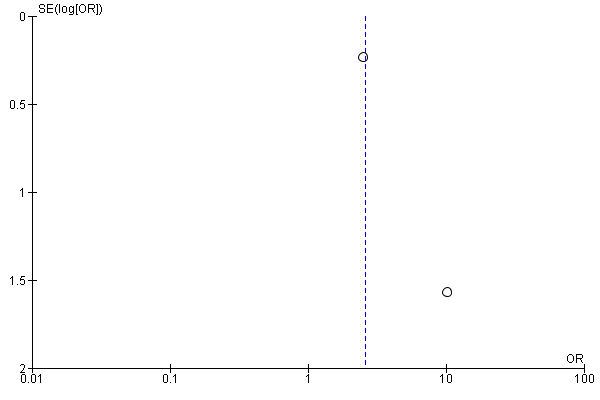


**Endoleaks at 4 years**


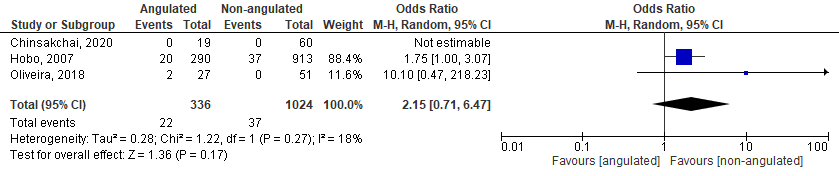


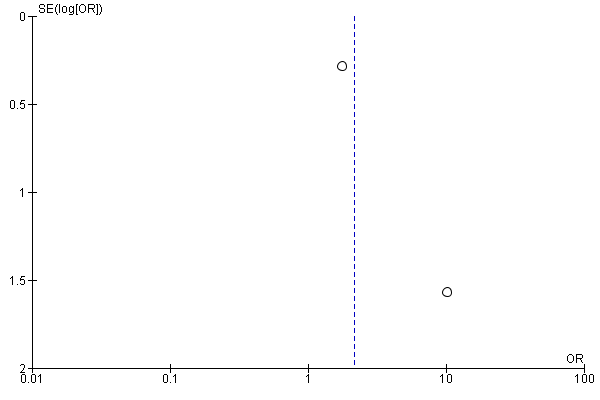


**Endoleaks at 5 years**


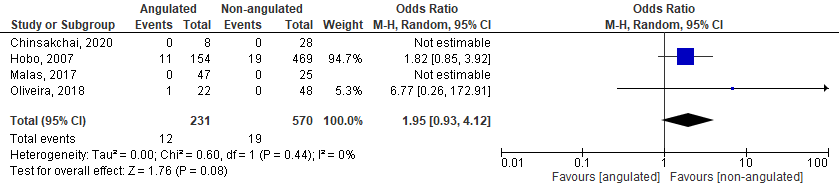


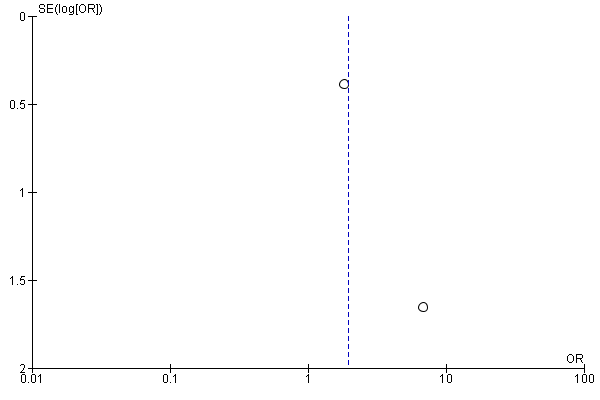


**Secondary procedure regarding proximal sealing**

**Secondary procedures at 30 days**


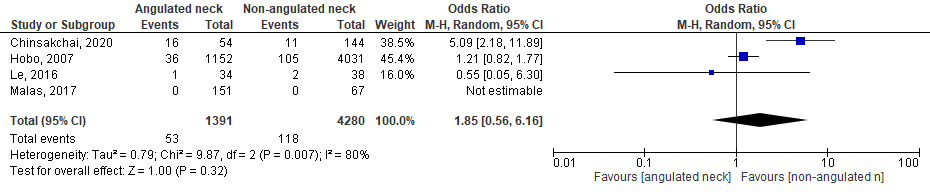


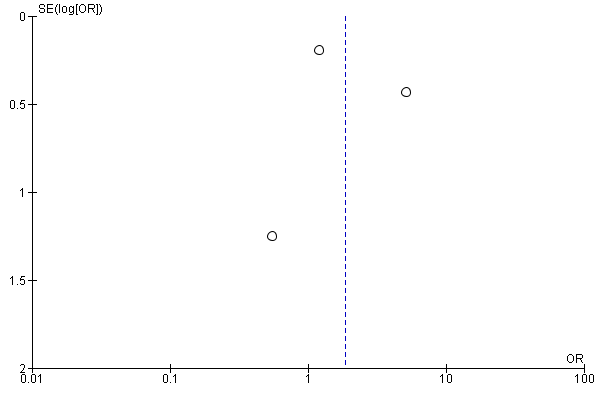


Secondary procedures at 1 year


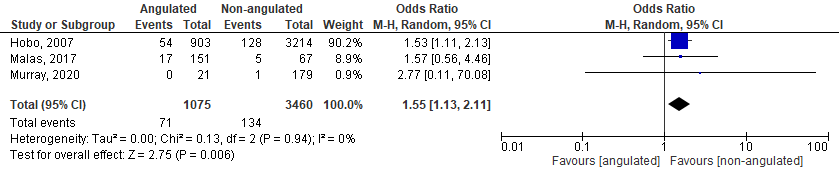


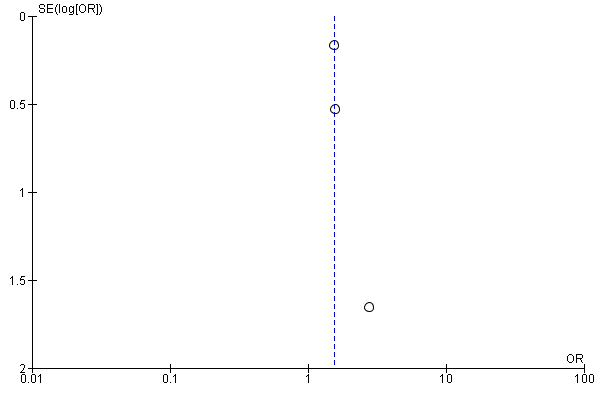


Secondary procedures 3 years


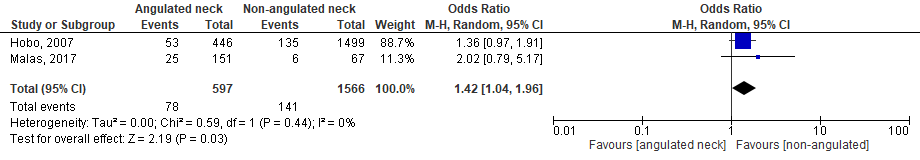


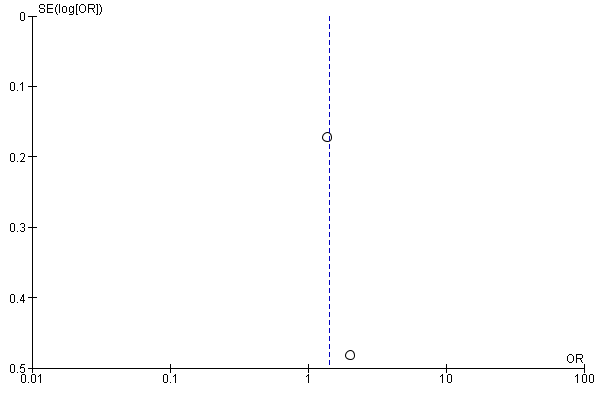


**Migration at 30 days**


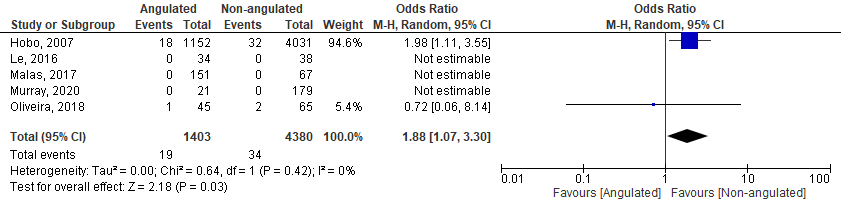


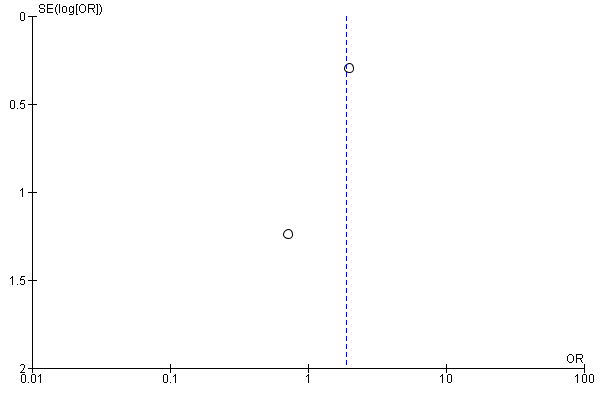


**Migration at 6 months**


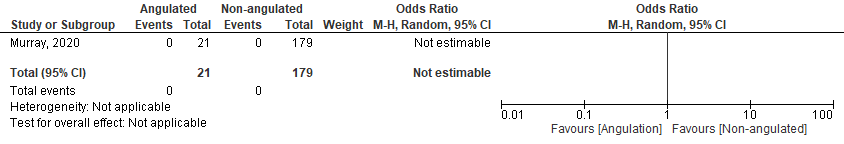


**Migration at 1 year**


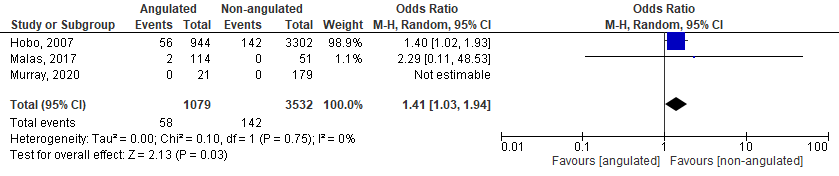


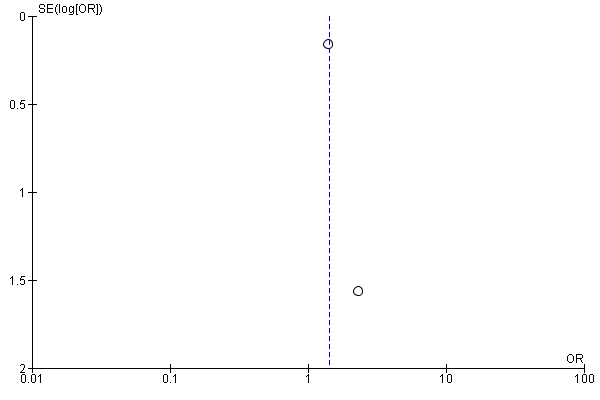


**Migration at 2 years**


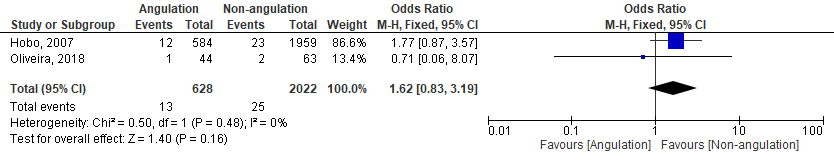


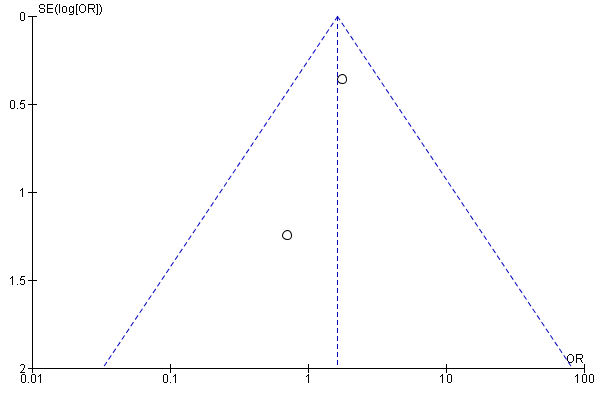


**Migration at 3 years**


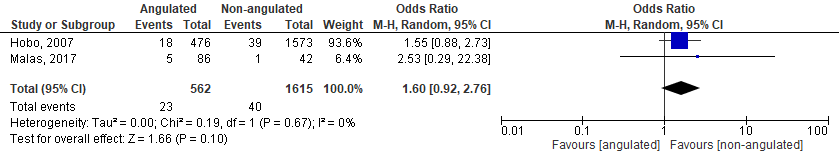


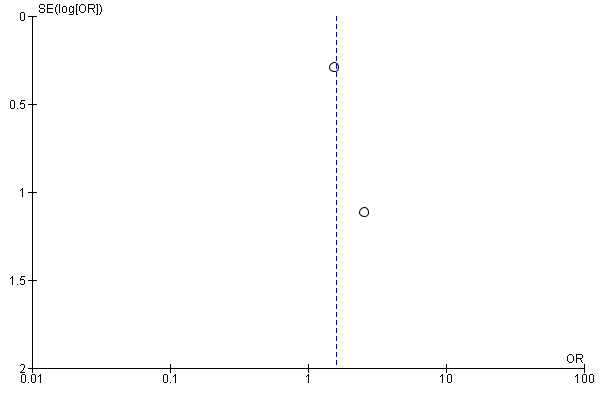


**Migration at 5 years**


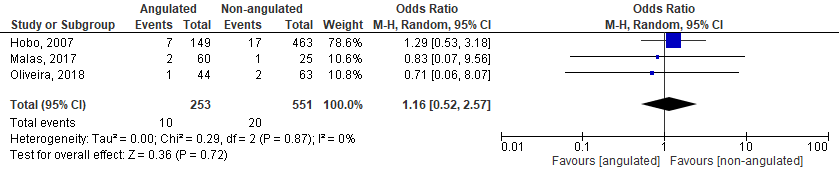


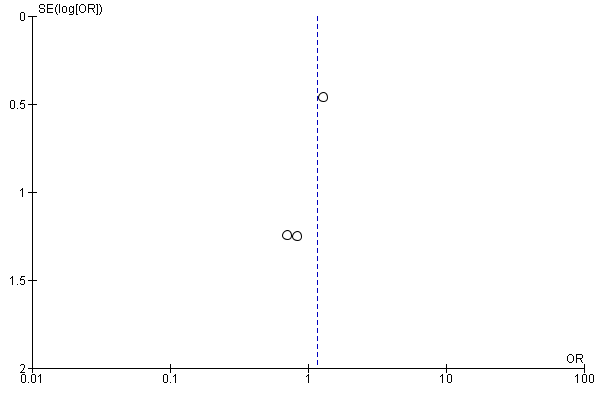


**Sac increase at 1 year**


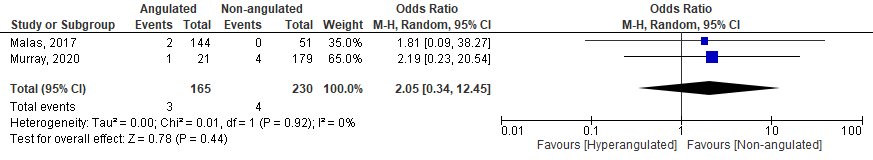


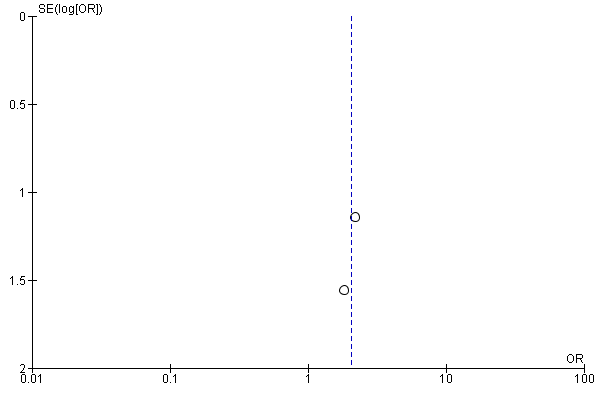


**Sac increase at 3 years**


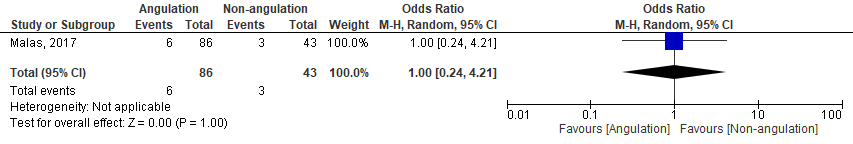


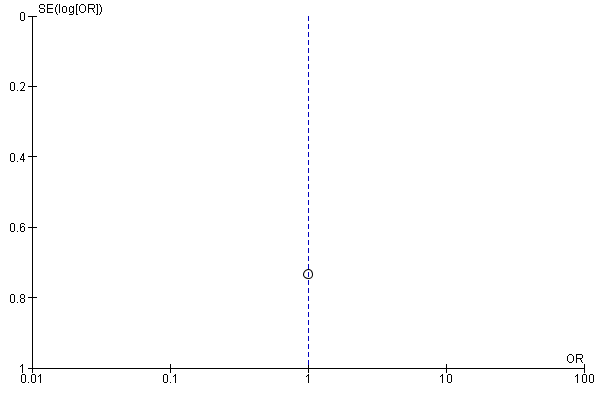


**Sac increase at 5 years**


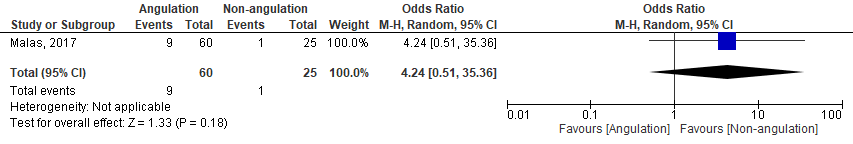


**Sac increase 5 years** 🡪 only 1 study


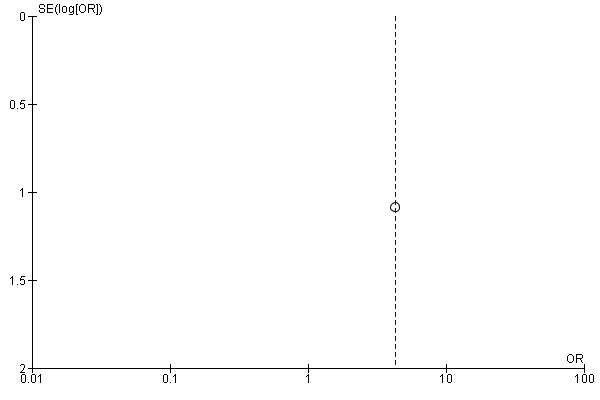


**Aneurysm rupture at 30 days**


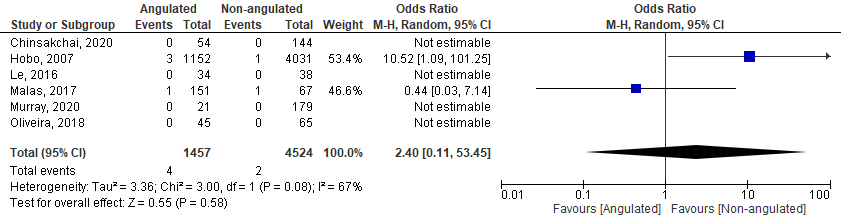


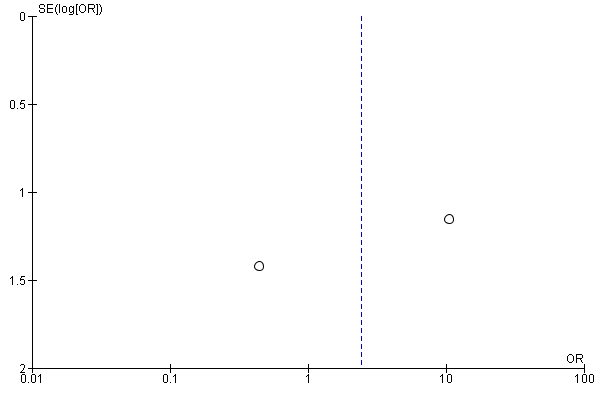


**Aneurysm rupture at 6 months**


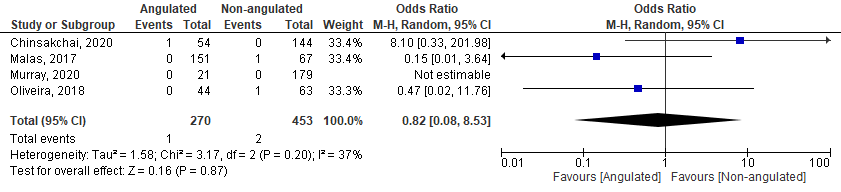


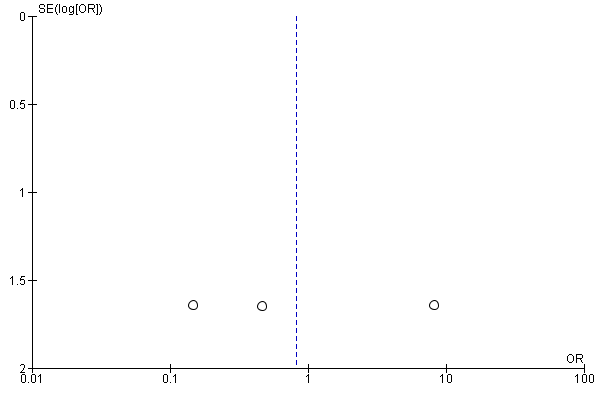


**Aneurysm rupture at 1 year**


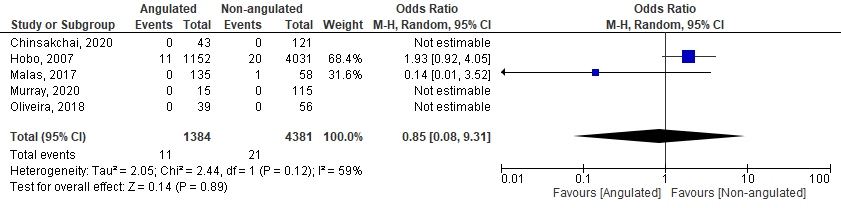


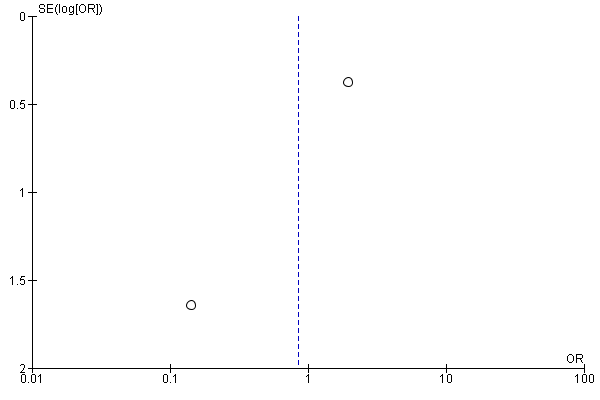


**Aneurysm rupture at 2 years**


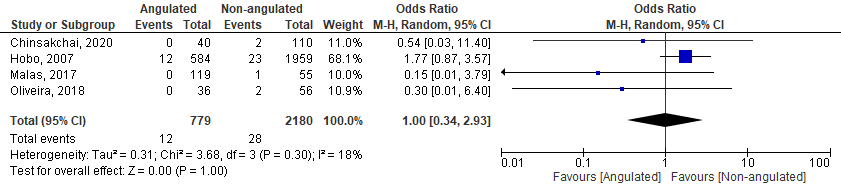


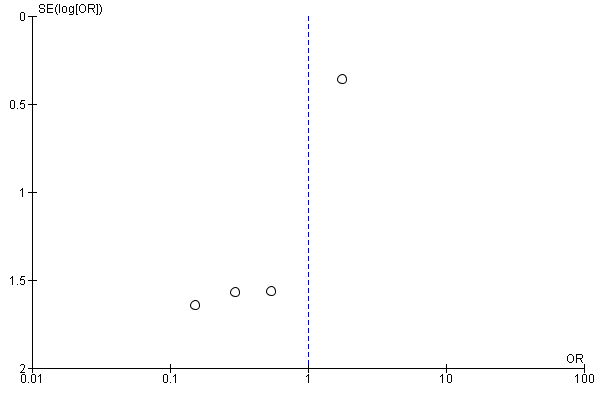


**Aneurysm rupture at 3 years**


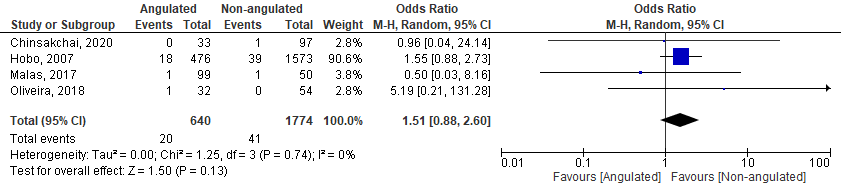


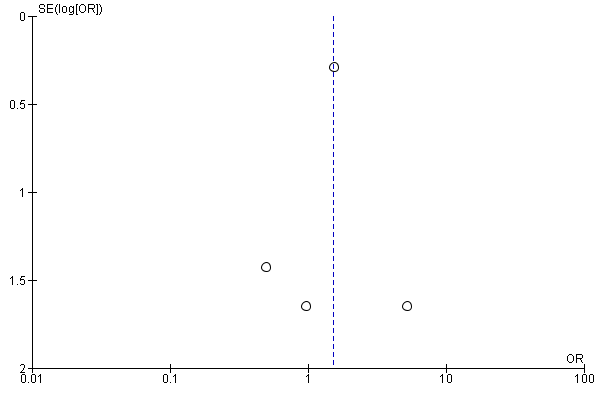


**Aneurysm rupture at 4 years**


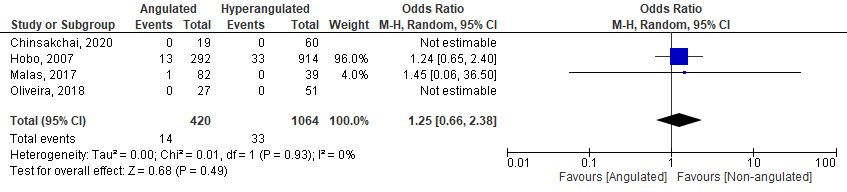


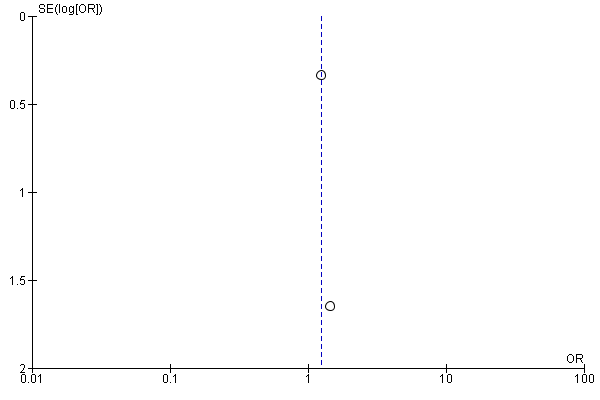


**Aneurysm rupture at 5 years**


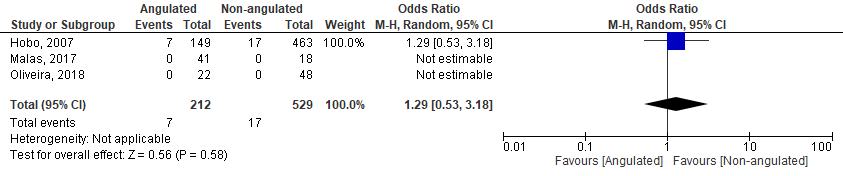


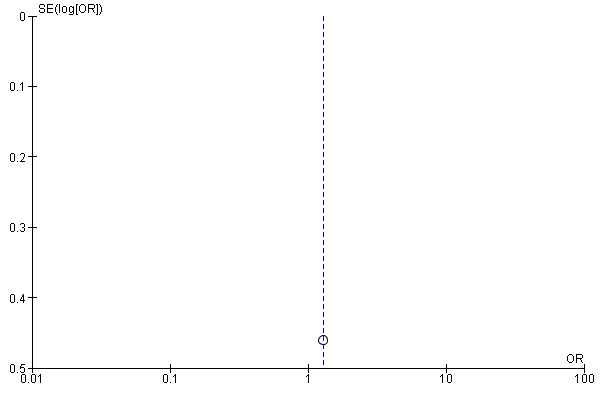


**Aneurysm related death at 30 day**


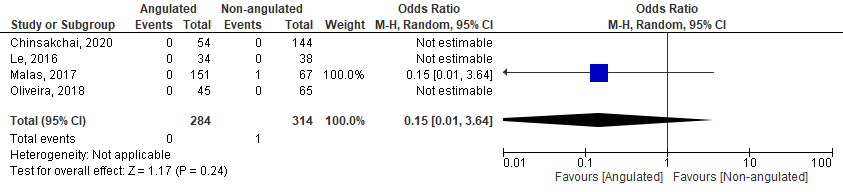


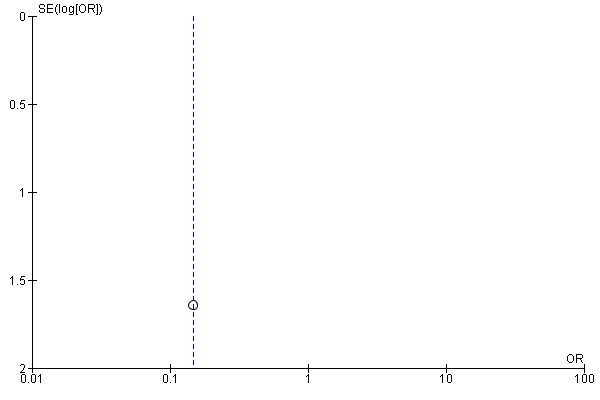


**Aneurysm related death at 6 months**


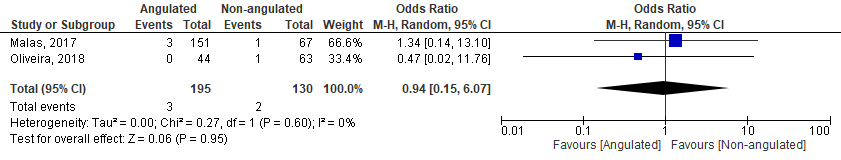


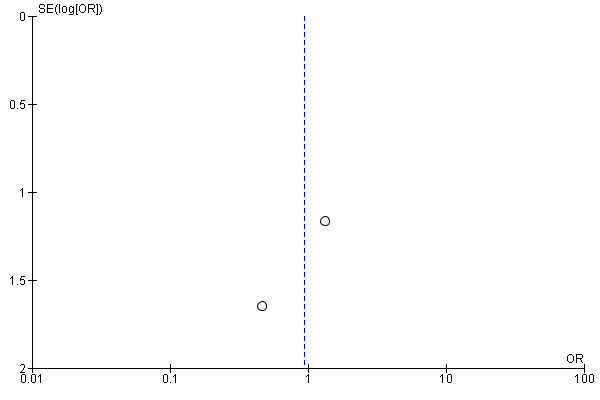


**Aneurysm related death at 1 year**


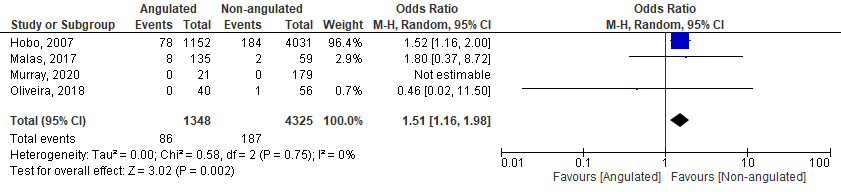


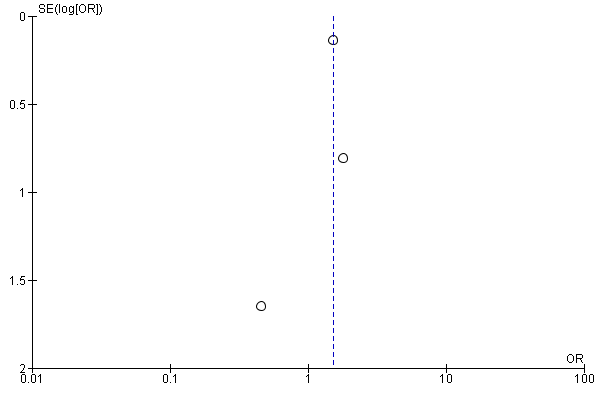


**Aneurysm related death at 2 years**


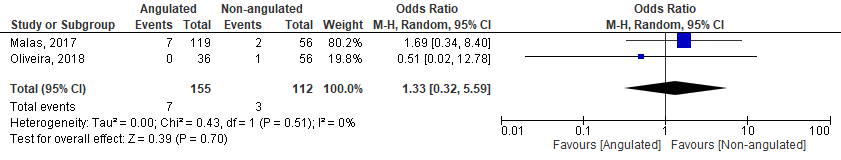


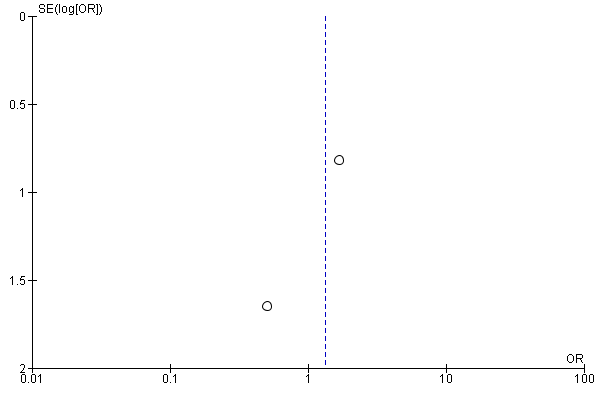


**Aneurysm related death at 3 years**


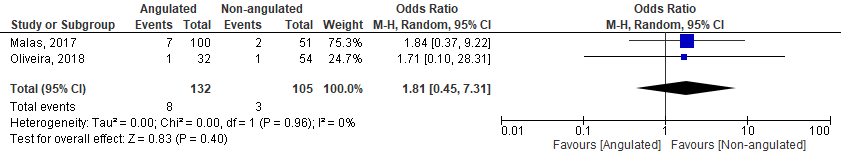


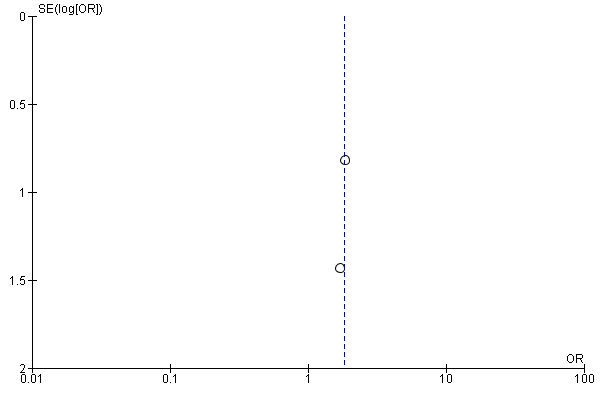


**Aneurysm related death 4 years**


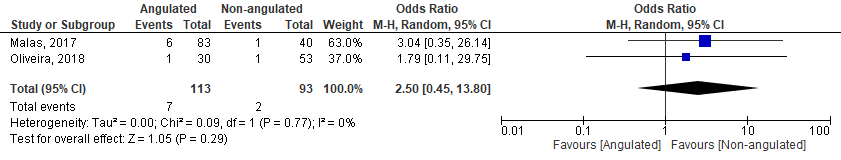


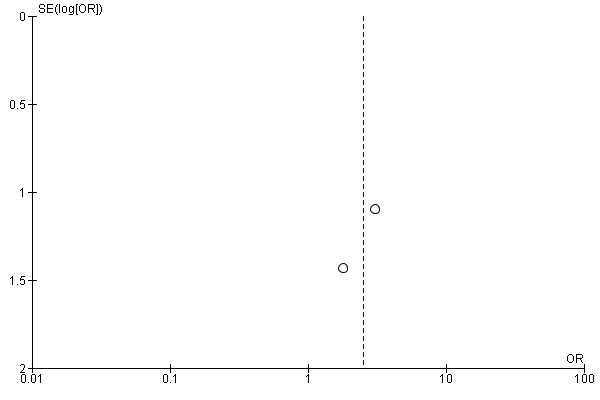


**Aneurysm related death at 5 years**


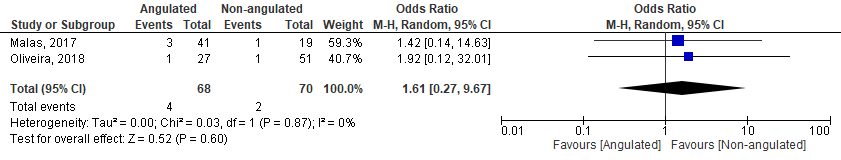


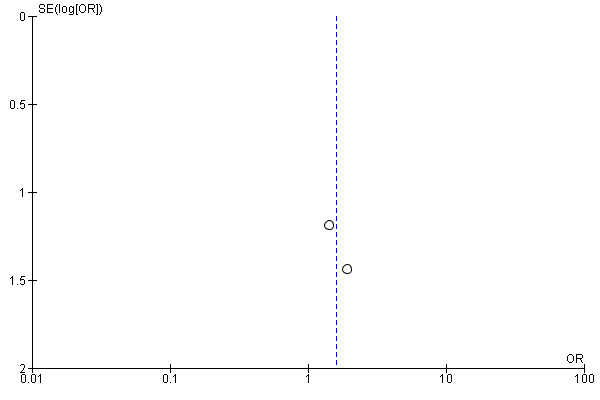


**All cause mortality at 30 days**

**
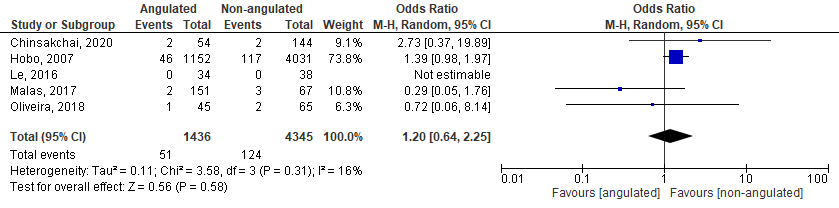
**

**
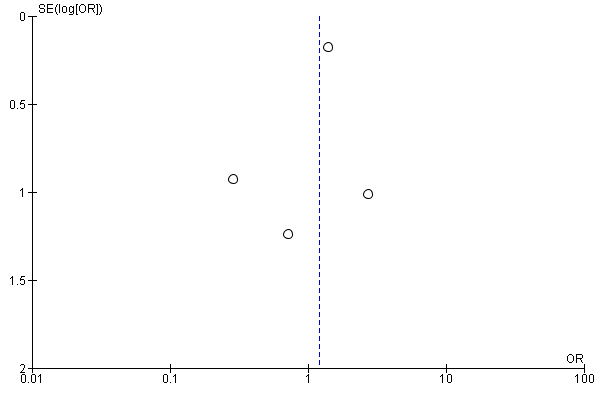
**

**All-cause mortality at 6 months**


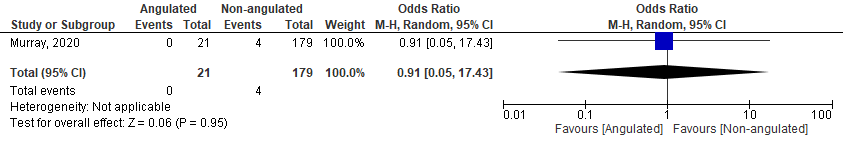


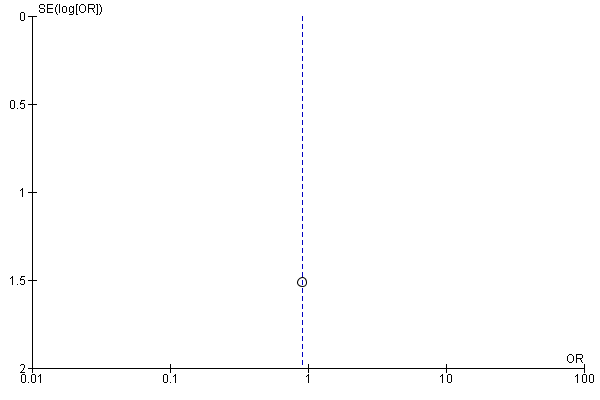


**All-cause mortality at 1 year**


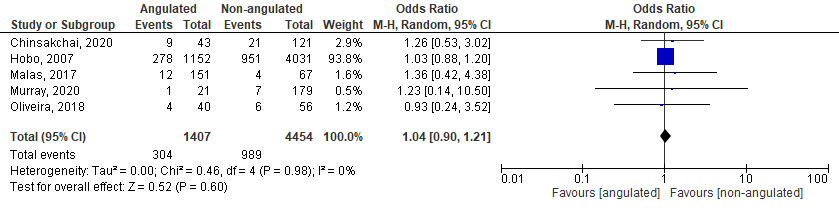


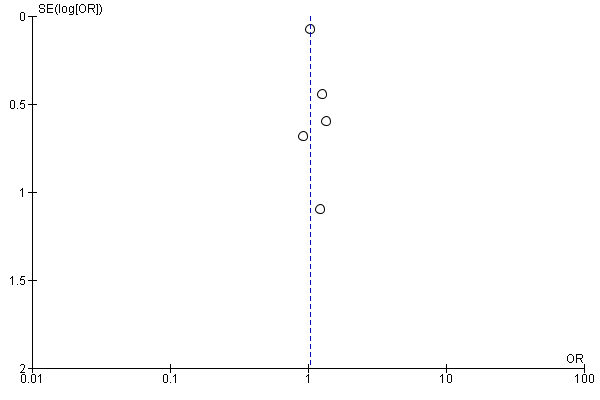


**All-cause mortality at 2 years**


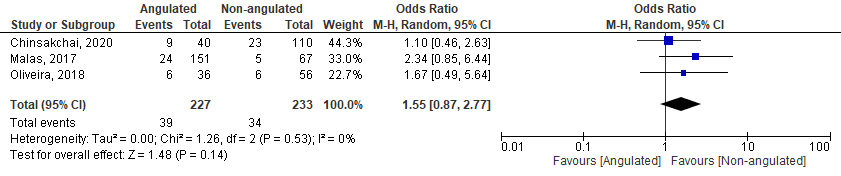


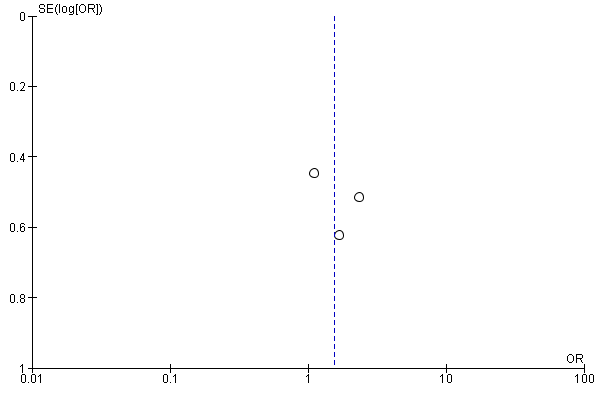


**All-cause mortality at 3 years**


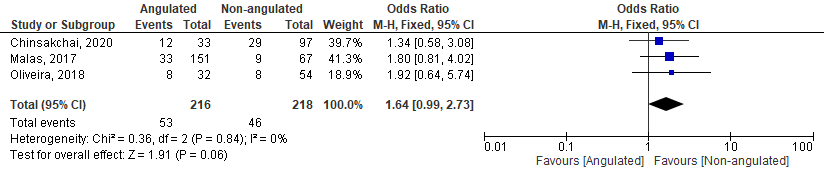


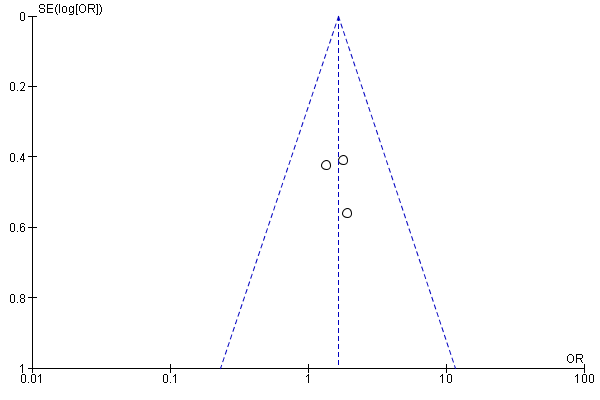


**All-cause mortality 4 years**


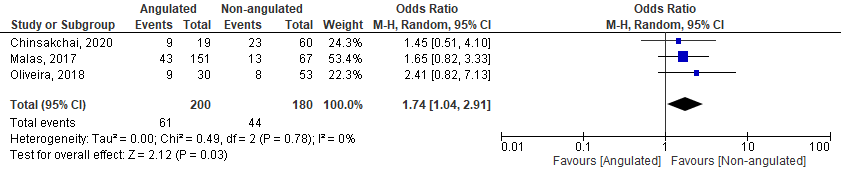


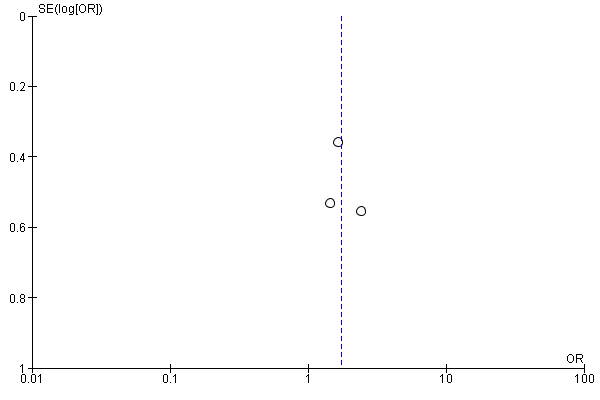


**All-cause mortality at 5 years**


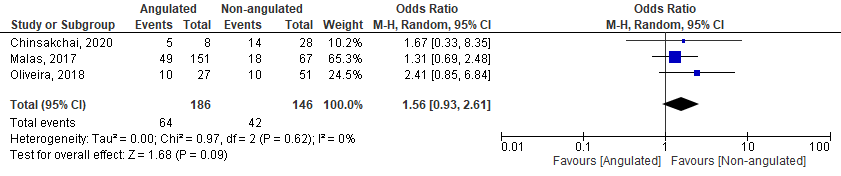


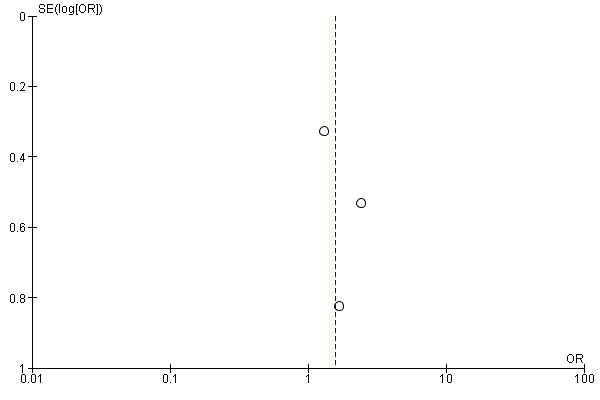


**Technical success**


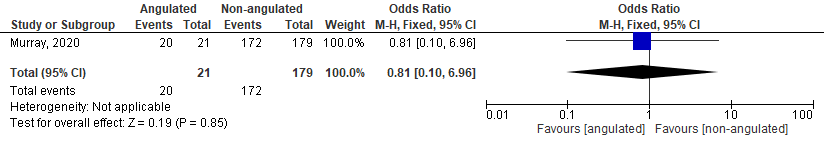


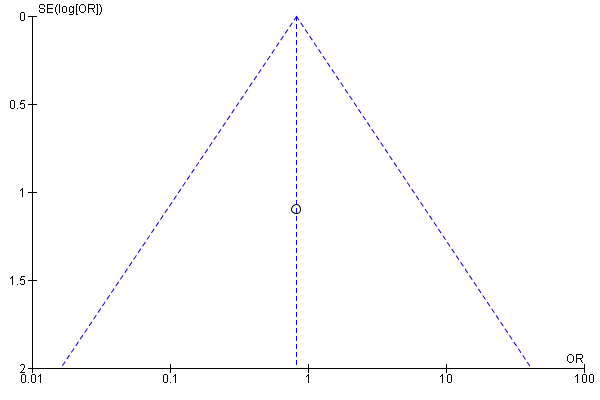

Supplement: S1 Dataset — (DOCX) [file pone.0264327.s007.docx]
